# Endemic Norovirus Infections in Children, Ho Chi Minh City, Vietnam, 2009–2010

**DOI:** 10.3201/eid1906.111862

**Published:** 2013-06

**Authors:** Phan Vu Tra My, Corinne Thompson, Hoang Le Phuc, Pham Thi Ngoc Tuyet, Ha Vinh, Nguyen Van Minh Hoang, Pham Van Minh, Nguyen Thanh Vinh, Cao Thu Thuy, Tran Thi Thu Nga, Nguyen Thi Thu Hau, James Campbell, Nguyen Tran Chinh, Tang Chi Thuong, Ha Manh Tuan, Jeremy Farrar, Stephen Baker

**Affiliations:** Oxford University Clinical Research Unit, Ho Chi Minh City, Vietnam (P.V. Tra My, C. Thompson, N.V.M. Hoang, P.V. Minh, N.T. Vinh, C.T. Thuy, T.T.T. Nga, J. Campbell, J. Farrar, S. Baker);; Oxford University, Oxford, UK (C. Thompson, J. Campbell, J. Farrar, S. Baker);; Children’s Hospital 1, Ho Chi Minh City (H.L. Phuc, T.C. Thuong);; Children’s Hospital 2, Ho Chi Minh City (P.T.N. Tuyet, N.T.T. Hau, H.M. Tuan);; Hospital for Tropical Diseases, Ho Chi Minh City (H. Vinh, N.T. Chinh);; The London School of Hygiene and Tropical Medicine, London, UK (S. Baker)

**Keywords:** norovirus, viruses, diarrhea, gastroenteritis, risk factors, symptomatic, asymptomatic, children, pediatric, Vietnam

## Abstract

We performed a case–control investigation to identify risk factors for norovirus infections among children in Vietnam. Of samples from 1,419 children who had diarrhea and 609 who were asymptomatic, 20.6% and 2.8%, respectively, were norovirus positive. Risk factors included residential crowding and symptomatic contacts, indicating person-to-person transmission of norovirus.

Norovirus (NoV) is a leading cause of acute gastroenteritis in children <5 years of age (*1*). The epidemiology of NoV in industrialized countries has been intensively investigated, yet the contribution of this pathogen to the effects of diarrheal disease in low- and middle–income countries is not well characterized (*1**,**2*). Gaining insight into the epidemiology of NoV infections of children in such countries is essential for disease control, particularly considering that several vaccine candidates are in advanced-stage clinical trials (*3*). To address the lack of data on risk factors for endemic NoV infections in low-income countries, we conducted a prospective case–control study among hospitalized children in a major urban location in southern Vietnam.

## The Study

This study was conducted in 3 hospitals (Children’s Hospital 1, Children’s Hospital 2, and the Hospital for Tropical Diseases) in Ho Chi Minh City, Vietnam, during May 2009–December 2010. Written informed consent from a parent or legal guardian was mandatory for participation. Children <5 years of age who resided in Ho Chi Minh City, who had acute diarrhea on admission (≥3 loose stools or ≥1 bloody loose stool within a 24-hour period), and were given no antimicrobial drug treatment 3 days before hospitalization, were invited to participate during May 2009–April 2010. To collect control data, during March–December 2010, we enrolled children who were attending outpatient and inpatient clinics in the nutrition or gastroenterology departments for routine health checks or conditions unrelated to gastroenteritis. Children in this control group met the same demographic criteria, did not have diarrhea, and had not received antimicrobial drugs during the preceding 3 weeks. 

Stool specimens were collected from case-patients on the day of admission (n = 1,419) and from control participants while they were attending the clinic (n = 609). All stool samples were cultured by using classic microbiologic methods to detect *Shigella, Salmonella*, *Campylobacter,* and *Yersinia* spp. and were microscopically examined for *Entamoeba, Cryptosporidium,* and *Giardia* spp. Methods are described in the Technical Appendix. Conventional reverse transcription PCR was performed on RNA extracted from stool samples to detect rotavirus (*4*) and NoV genogroups I (GI) and II (GII) (*5*), followed by direct sequencing of the amplicons for genotyping.

After rotavirus (46.6%; 661/1,419), NoV was the second most common pathogen detected in symptomatic case-patients (20.6%; 293/1,419); diarrheal bacteria and parasites were cumulatively found in 14.5% (Technical Appendix). The prevalence of NoV was higher than in a pooled international estimate (*1*) and than in previous studies performed in Ho Chi Minh City (*6**–**8*), yet was lower than that found in a study conducted in northern Vietnam (*9*). The frequency of NoV detected in control participants was 2.8% (17/609), similar to a pooled international estimate (*1*). The majority of NoV-positive case-patients experienced nonbloody, nonmucoid watery diarrhea, vomiting, and fever. These symptoms were comparable to those in previous studies of diarrheal infections in children in Vietnam (*7**,**9*).

NoV was detected throughout the study period (Technical Appendix Figure). There was a positive linear correlation between NoV infections and monthly rainfall (R = 0.550, p = 0.029), but no similar correlation with temperature (range 22.1°C–37.8°C) (R = 0.308, p = 0.330). This association of NoV infections with the tropical rainy season may reflect differential transmission between different climatic regions because NoV infections are typically associated with the winter season in industrialized countries in temperate regions (*10*).

GII NoV was detected in 239 (99.1%) of 241 and 11 (73.3%) of 15 NoV-positive stool samples from the symptomatic and asymptomatic enrollees, respectively. The remaining children were infected with NoV GI (GI.3, GI.4, GI.5); 1 enrolled case-patient was infected with 2 genotypes: NoV GI.3 and GII.4. Of the GII strains, GII.4 was the most prevalent genotype, comprising 201 (84.1%) of the 239 samples. The next most prevalent was GII.3: 24 (10.0%) were identified in the symptomatic and asymptomatic groups. Other GII genotypes (GII.2, GII.6, GII.7, GII.9, GII.12, and GII.13) were found in <3% of NoV-positive samples.

Socioeconomic and behavioral data were obtained from all enrollees by using a questionnaire and analyzed by using Stata Version v9.2 (StataCorp LP, www.stata.com) (Table 1). We used χ^2^ and Fisher exact tests to compare proportions between groups and Mann-Whitney U tests for nonparametric data. Univariate analyses were performed to assess factors associated with symptomatic NoV infections. Factors found to be significantly associated with infection in the univariate analysis, in addition to a-priori factors of age, sex, and income level, were then included in a multivariate logistic regression model to simultaneously control for confounding effects. Two-sided p values ≤0.05 were considered significant throughout (Table 2).

**Table 1:** Baseline characteristics of NoV-positive and NoV-negative case-patients and control participants, Vietnam, 2009–2010*

Ch*a*racteristic	Case-patients		Controls

NoV positive, n = 241	NoV negative, n = 1,126	NoV positive, n = 15	NoV negative, n = 592
Male sex	147 (61.0)	724 (64.3)		8 (53.3)	314 (53.0)
Mean age, mo (range)	13.3 (2–45)	15.8 (1–59)		15.8 (2.3–52)	16.8 (0–60)
Age groups, mo					
<6	24 (10.0)	165 (14.7)		3 (20.0)	98 (16.6)
7–12	102 (42.3)	375 (33.3)		4 (26.7)	208 (35.1)
13–18	76 (31.5)	245 (21.8)		3 (20.0)	113 (19.1)
19–24	25 (10.4)	147 (13.1)		3 (20.0)	56 (9.5)
24–60	14 (5.8)	194 (17.2)		2 (13.4)	117 (19.8)
Poor Z score†	18 (7.5)	73 (6.5)		1 (6.7)	75 (12.7)
Breastfed	187 (77.6)	790 (70.2)		11 (73.3)	452 (76.4)
Daily activity					
Day care/nursery school	30 (12.5)	194 (16.4)		4 (26.7)	89 (15.2)
Home	211 (87.6)	938 (83.6)		11 (73.3)	498 (84.8)

*Values are no. case (%) unless otherwise specified. Case-patients indicate patients who had diarrhea; controls indicate asymptomatic (diarrhea-free) children. Study dates span May 2009–December 2010. NoV, norovirus; WHO, World Health Organization. †Weight-for-age Z score calculated based on WHO Child Growth Standards guidelines (www.who.int/childgrowth/standards/technical_report/en**/**); Z score below −2 was considered to indicate that a child was malnourished.

**Table 2:** Univariate and multivariate analysis of risk factors for symptomatic NoV infections, Vietnam, 2009–2010*

Risk factor	NoV-positive case-patients	NoV-negative control participants	OR	95% CI	aOR	95%CI
Mean age, mo (range)	13.3 (2–45)	16.8 (0–60)	**0.97**	**0.96**–**0.99**	**0.96**	**0.94**–**0.98**
Male sex (%)	147 (61.0)	314 (53.0)	**1.38**	**1.0**–**1.9**	1.38	0.9–2.0
Poor Z-score	18 (7.5)	75 (12.7)	**0.56**	**0.3**–**0.9**	0.61	0.3–1.1
Low income†	150 (62.2)	335 (56.6)	1.26	0.9–1.7	0.89	0.6–1.3
≥5 adults in hh	72 (29.9)	158 (26.7)	1.17	0.8–1.6	NI	x
≥3 children in hh	36 (14.9)	58 (9.8)	**1.62**	**1.0**–**2.5**	**1.70**	**1.0**–**2.9**
Refrigerator in hh	187 (77.6)	506 (85.5)	**0.59**	**0.4**–**0.9**	0.73	0.5–1.2
Consumes market food	201 (84.1)	345 (58.4)	**3.77**	**2.6**–**5.5**	**4.99**	**3.1**–**7.9**
Household water source						
Pipeline‡	132 (54.8)	347 (58.6)	1.00	NA	NI	NI
Well	96 (39.8)	220 (37.2)	1.15	0.8–1.6	NI	NI
Other§	13 (5.4)	25 (4.2)	1.37	0.7–2.8	NI	NI
Drinking water source	NI	NI	NI	NI	NI	NI
Pipeline‡	116 (48.1)	334 (56.4)	1.00	NA	1.00	NA
Bottled water	69 (28.6)	122 (20.6)	**1.63**	**1.1**–**2.3**	**2.18**	**1.4**–**3.4**
Well	42 (17.4)	109 (18.4)	1.11	0.7–1.7	0.94	0.6–1.5
Other§	14 (5.8)	1.49	0.8–2.9	0.25	1.45	0.6–3.2
Toilet type						
Indoor‡	213 (90.6)	446 (75.9)	1.00	NA	1.00	NA
Outdoor	22 (9.4)	142 (24.2)	**0.32**	**0.2**–**0.5**	**0.22**	**0.1**–**0.4**
Hand washing¶	130 (85.0)	291 (89.5)	0.66	0.4–1.2	NI	NI
Attends day care/nursery school	30 (12.5)	89 (15.2)	0.80	0.5–1.2	NI	NI
Contact with symptomatic persons	38 (16.5)	8 (1.4)	**14.23**	**6.5**–**31.0**	**26.14**	**10.4**–**65.9**
Rural residence#	36 (14.9)	75 (12.7)	1.21	0.8–1.9	NI	NI

*Values are no. case (%) unless otherwise specified. Values in **boldface** indicate statistical significance at p≤0.05. NoV, norovirus; OR, odds ratio; aOR, adjusted OR; NA, not applicable; NI, not included in multivariable analysis; hh, household.  †Classified as making less than the Gross National Income ($232/mo) according to World Bank (http://data.worldbank.org/indicator/NY.GNP.PCAP.CD). ‡Reference group. §Rain water, water from a truck provided by the government, or other water source. ¶Washing of children’s hands, either by an adult or the child, after the child uses the toilet. #Binh Chanh, Can Gio, Cu Chi, Hoc Mon, and Nha Be districts.

NoV infections are commonly associated with outbreaks in enclosed environments (*2*), yet we found attendance in daycare centers and nursery schools was not common; the majority of children remained at home during the day. However, several factors were significantly and independently associated with symptomatic NoV infections. Demographic risk factors included younger age (in months) (adjusted odds ratio [aOR] 0.96, 95% CI 0.94–0.98, p<0.001) and household crowding (≥3 children in the house) (aOR 1.70, 95% CI 1.0–2.9, p = 0.052). Living in a household where food was regularly purchased from outdoor markets added a significant risk (aOR 4.99, 95% CI 3.1–7.9, p<0.001). Unpredictably, we found that consuming bottled water, rather than pipeline water (aOR 2.18, 95% CI 1.4–3.4, p< 0.001), was a risk factor and did not correlate with household income. However, those drinking municipal water also reported boiling or filtering water before consumption, and those drinking bottled water did not. This association suggests that bottled water in this location may be of poor quality. A further unexpected finding was the protective nature of outdoor toilets (aOR 0.22, 95% CI 0.1–0.4, p<0.001), which may be a result of the sterilizing capabilities of sunlight or of containing fecal contamination outside the residence, possibly protecting children during the period of infancy before they can use toilets. We found that the greatest risk factor for symptomatic NoV infections (aOR 26.14, 95% CI 10.4–65.9, p<0.001) was contact with a person who recently had a diarrheal infection. This finding is consistent with previous investigations showing that person-to-person transmission is predominant during sporadic outbreaks (*11**–**14*).

This study has several limitations. First, passive case detection limits generalizability because health care–seeking behavior may depend on disease severity and income in this setting. Second, the control participants may not be entirely representative of the population from which the case-patients arose because a large proportion of the control participants were visiting the hospital for nutritional advice, which may have an effect on diarrheal disease risk (*15*). Yet, a limited sensitivity analysis comparing NoV-positive case-patients to NoV-negative control participants and NoV-negative case-patients to NoV-negative control participants demonstrated several differences in risk factors, suggesting that the identified risk factors are associated with NoV rather than health care–seeking behavior (Appendix Table 2).

## Conclusions

This epidemiologic investigation showed that 20.6% of hospitalized children with acute diarrhea in Ho Chi Minh City tested positive for NoV, compared with 2.8% of diarrhea-free control participants. We conclude that young age, residential crowding, use of bottled water, and recent contact with a symptomatic individual are key risk factors for symptomatic NoV infection in this location. Because most children did not attend day care, potential preventative measures for NoV infection in Ho Chi Minh City should be focused on improving local hygiene standards to prevent person-to-person transmission within the home.

Technical Appendix Proportion of pathogens identified related to diarrheal illness, proportion of diarrheal cases that were positive for norovirus (single-organism infection) by month compared to average monthly rainfall accumulation, and microbiologic screening methods for diarrheal bacteria and parasites.
