# Supplementary material for: Endemic Norovirus Infections in Children, Ho Chi Minh City, Vietnam, 2009–2010
Source: Emerg Infect Dis. 2013 Jun;19(6):977–80. doi: 10.3201/eid1906.111862 (PMC3713809; doi:10.3201/eid1906.111862)
Supplement: Technical Appendix — Proportion of pathogens identified related to diarrheal illness, proportion of diarrheal cases that were positive for norovirus (single-organism infection) by month compared to average monthly rainfall accumulation, and microbiologic screening methods for diarrheal bacteria and parasites. [file 11-1862-Techapp-s1.pdf]

Article DOI: http://dx.doi.org/10.3201/eid1906.111862

Endemic Norovirus Infections in Children, Ho Chi Minh City, Vietnam, 2009–2010

Technical Appendix

Technical Appendix Table. Proportion of pathogens identified related to diarrheal illness, Vietnam, 2009–2010

Norovirus infections	N = 293 (%)
Single-pathogen infections	241 (82.3)
Dual-pathogen infections	52 (17.7)
Rotavirus	32 (10.9)
Bacteria	18 (6.1)
Campylobacter spp.	6 (2.0)
Salmonella spp.	8 (2.7)
Shigella spp.	3 (1.0)
Aeromonas spp.	1 (0.3)
Mixed viral/bacterial	2 (0.7)
Rotavirus/*Campylobacter* spp	1 (0.3)
Rotavirus/*Salmonella* spp	1 (0.3)

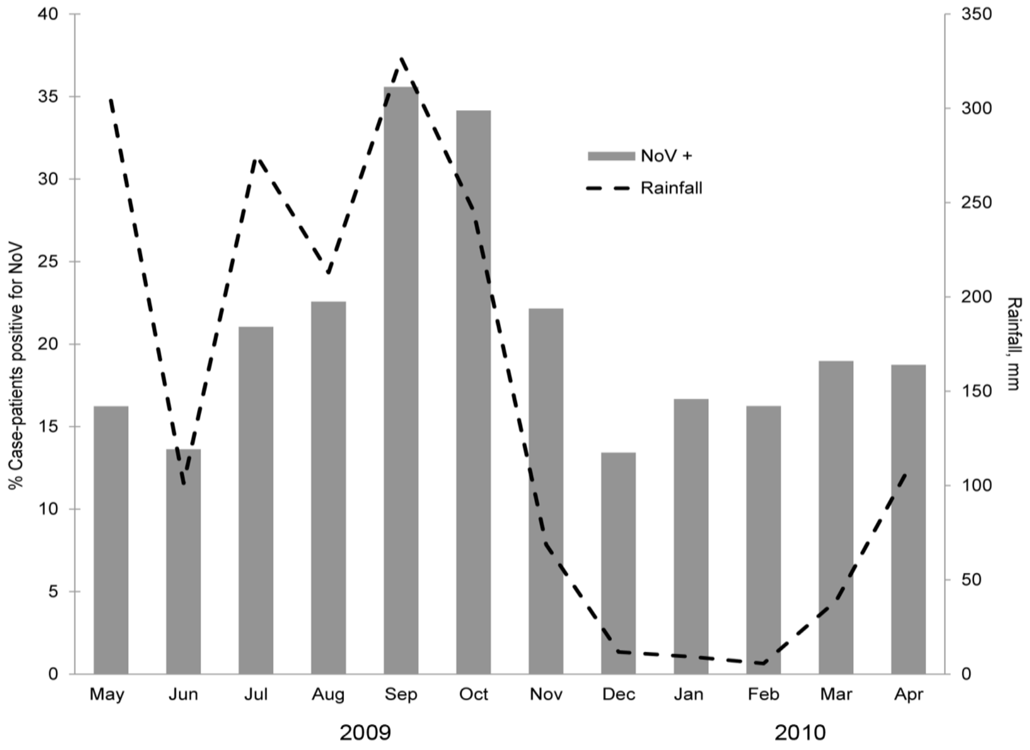

Technical Appendix Figure. Proportion of diarrheal cases that were positive for norovirus (NoV) (single-organism infection) (dark bars) by month compared to average monthly rainfall accumulation measured in mm (black line) in Ho Chi Minh City, Vietnam, 2009–2010. Climatic data were obtained from Vietnam Southern Regional Hydro-Meteorological Station.

Microbiologic Screening Methods for Diarrheal Bacteria and Parasites

Stool specimens were cultured on blood agar, MacConkey agar, xylose-lysine-deoxycholate agar, selenite broth, and *Campylobacter* media (Oxoid, Basingstoke, UK) to isolate *Shigella* spp., *Salmonella* spp., *Campylobacter* spp., *Yersinia* spp., *Pleisiomonas* spp., and *Aeromonas* spp., according to guidelines of Clinical and Laboratory Standards Institute. Apart from *Campylobacter* plates, which were incubated micro-aerophilically at 42°C, all media were incubated at 37°C overnight. Colonies of suspected pathogens were subcultured onto nutrient agar for purity, and bacterial identity was confirmed by API 20E and serotyping where applicable (bioMérieux, Paris, France). For parasite microscopy, a fresh smear of stool specimen was mixed with phosphate-buffered saline; 10 µL of the resulting stool sample was microscopically examined at 400 times magnification for *Entamoeba histolytica*, *Cryptosporidium* spp., and *Giardia lamblia* cysts.

Norovirus Genotyping

All NoV-positive PCR amplicons were purified by using QIAGEN PCR purification kits (QIAGEN, Hilden, Germany) and subjected to direct sequencing by using the same primers used for primary amplification. The PCR primers amplify a region of 330bp of NoVgenogroup I and 387bp of NoVgenogroup II (*6*). Direct sequencing was performed by using the BigDye Terminator Cycle Sequencing kit (Applied Biosystems, https://products.appliedbiosystems.com/ab/en/US/adirect/ab?cmd=catNavigate2&catID=601943) and the DNA sequences were generated by using an ABI Prism 3130xl Genetic Analyzer (Applied Biosystems, https://products.appliedbiosystems.com/ab/en/US/adirect/ab?cmd=catNavigate2&catID=602103) according to the manufacturer’s recommendations. Resulting DNA sequences were assembled by using DNA Baser Sequence Assembler v3.0.17 (HeracleBiosoft, Pitesti, Romania). NoV genotypes were assigned by using the online Norovirus Automated Genotyping Tool (http://www.rivm.nl/mpf/norovirus/typingtool).
